# LINC00261 Suppresses Cisplatin Resistance of Esophageal Squamous Cell Carcinoma Through miR-545-3p/MT1M Axis

**DOI:** 10.3389/fcell.2021.687788

**Published:** 2021-07-15

**Authors:** Lijun Wang, Xiaojun Wang, Pengwei Yan, Yatian Liu, Xuesong Jiang

**Affiliations:** Department of Radiation Oncology, Jiangsu Cancer Hospital, Jiangsu Institute of Cancer Research, The Affiliated Cancer Hospital of Nanjing Medical University, Nanjing, China

**Keywords:** LINC00261, miR-545-3p, MT1M, DDP resistance, ESCC

## Abstract

To improve the survival rate and cure rate of patients, it is necessary to find a new treatment scheme according to the molecular composition of (ESCC) in esophageal squamous cell carcinoma. Long non-coding RNAs (lncRNAs) regulate the progression of ESCC by various pathophysiological pathways. We explored the possible function of the lncRNA LINC00261 (LINC00261) on cisplatin (DDP) resistance of ESCC and its relative molecular mechanisms. In the study, we found that LINC00261 was downregulated in ESCC tissues, cell lines, and DDP-resistant ESCC patients. Besides, overexpression of LINC00261 not only inhibited cell proliferation, and DDP resistance but also promotes cell apoptosis. Further mechanistic research showed that LINC00261 sponged miR-545-3p which was negatively correlated with the expression of LINC00261. In addition, functional experiments revealed that upregulation of miR-766-5p promoted proliferation and enhanced DDP resistance. Subsequently, MT1M was testified to be the downstream target gene of miR-545-3p. Rescue experiments revealed that overexpression of MT1M largely restores miR-545-3p mimics-mediated function on ESCC progression. Our results demonstrate that the LINC00261 suppressed the DDP resistance of ESCC through miR-545-3p/MT1M axis.

## Introduction

Esophageal carcinoma (EC) serves as one of the most common malignant tumors with strong aggressiveness and poor prognosis, and it is the sixth leading cause of cancer-related death worldwide (Adenis et al., 2015). According to the latest cancer epidemiology, the incidence and fatality of esophageal cancer in China ranks fifth and fourth, respectively (Zhang et al., 2016; Zuo et al., 2016). Pathologically, more than 90% of EC in China is esophageal squamous cell carcinoma (ESCC) (Chen et al., 2016; Bray et al., 2018). Despite the great progress in the treatment of EC in recent years, the prognosis of ESCC was still very poor, with a 5-year survival rate of less than 10% (Ohashi et al., 2015). Four reasons contribute to the poor prognosis of EC which are (1) the detection time of ESCC is later than other cancers; (2) the lymph node metastasis is earlier; (3) surgical treatment is difficult to eradicate; (4) often accompanied by recurrence; and (5) metastasis after treatment (Okugawa et al., 2015; Jung et al., 2020). Besides, drug resistance is also an important reason for the failure of chemotherapy and poor prognosis in patients with ESCC. Clinical data shows that more than 90% of the deaths of patients with malignant tumors are related to chemotherapy resistance (Luqmani, 2005; Vadlapatla et al., 2013). Therefore, understanding the molecular mechanisms of ESCC proliferation, metastasis, and drug resistance has profound scientific significance and value for improving treatment methods and strategies and predicting the clinical prognosis (Lou et al., 2019).

Long non-coding RNA (lncRNA) is a kind of regulatory RNA whose transcriptional length exceeds 200 nucleotides and has no protein coding ability (Geisler and Coller, 2013). LncRNA has been proven to be closely related to the occurrence and development of tumors (Xiao et al., 2019). LncRNA regulates the expression of coding genes in many aspects such as transcriptional regulation, post-transcriptional regulation, and epigenetics, and thus plays a corresponding role in the occurrence, development, proliferation, metastasis, invasion, and drug resistance of tumors (Cheng et al., 2020; Liang et al., 2020). Currently, the role of lncRNA in tumors is a research hotspot, including lung cancer, liver cancer and bladder cancer (Chen et al., 2017, 2020; Gao et al., 2020). In addition, the role of lncRNAs in ESCC has also been extensively reported. For example, lncRNA LOC440173 promotes the progression of ESCC by modulating the miR-30d-5p/HDAC9 axis and the epithelial-mesenchymal transition (EMT) (Wang et al., 2020). In addition, lncRNA FOXD2-AS1 promotes DDP-resistance in ESCC by the miR-195/Akt/mTOR PATH (Liu H. et al., 2020). Lin et al. (2019) proved that LINC00261 is highly expressed in EC. Taken together, these studies imply that LINC00261 is involved in EC, but the specific role of LINC00261 in ESCC is still unclear. This study explored the molecular mechanism of LINC00261 on the biological processes of ESCC proliferation, apoptosis, and DDP-resistance from the cellular and molecular biology level, laying the foundation for ESCC targeted therapy and patient prognosis analysis.

## Materials and Methods

### ESCC Patients and Clinical Samples

Total 30 paired ESCC tumor tissues and adjacent non-tumor tissues were collected from Jiangsu Cancer Hospital. All patients did not get treatment. Patient data and samples are anonymized and obtained in accordance with ethical and legal guidelines. All participants had signed informed consent forms. This study was approved by the Ethics Committee of Jiangsu Cancer Hospital.

### Cell Culture and Cell Treatment

Two ESCC cell lines (TE-1 and ECA109) were purchased from the Chinese Academy of Sciences cell bank (Shanghai, China). All cells were cultured in RPMI-1640 containing contained with 10% fetal bovine serum (FBS) and incubated at 5% CO_2_ 37°C.

MiR-545-3p mimics and corresponding control NC-mimics, vectors for overexpression of LINC00261, MT1M and their negative control vector were obtained from the RiboBio (Guangzhou, China). These plasmids were transfected into cells by Lipofectamine 3000 (Thermo Fisher Scientific, United States) according to the protocols.

### Establishment of DDP−Resistant Cell Lines

DDP-resistant ESCC cells (TE-1/DDP and ECA109/DDP) were constructed by gradually increasing the concentration of DDP (Macklin, Shanghai, China) according to previous reports (Lin et al., 2019; Liu H. et al., 2020). Briefly, TE-1 and ECA109 were exposed to an initial DDP concentration of 0.2μmol/L in RPMI−1640 containing 10% FBS for 72 h. After washing three times with PBS, cells were cultured in DDP−free medium. Upon reaching 70–80% confluence, the cells were cultured with a higher concentration (10–20% increase per passage) of DDP. The above treatment was repeated until the concentration reached 10 mol/L.

### RT-qPCR

Total RNA was extracted from samples using the Trizol reagents (Takara, Otsu, Shiga, Japan) according to the manuals of manufacturers. The PrimeScriptTMII 1st Strand cDNA Synthesis Kit (Takara, Otsu, Shiga, Japan) was used to induced cDNA. Quantitative real-time quantitative PCR (qPCR) was performed using SYBR green main mixture in a 4800 real-time PCR instrument (Bio-RAD, CA, United States). U6 and β-actin were used as internal references. The 2^–ΔΔ*Ct*^ method was used to calculate the relative mRNA expression levels. The primers for RT-qPCR were shown in Table 1.

**TABLE 1:** Primer sequences used for RT-qPCR.

Genes		Primer sequences (5′–3′)
LINC00261	Forward	GTCAGAAGGAAAGGCCGTGA
x	Reverse	TGAGCCGAGATGAACAGGTG
MT1M	Forward	
	Reverse	
β-actin	Forward	AGCGAGCATCCCCCAAAGTT
	Reverse	GGGCACGAAGGCTCATCATT
miR-545-3p	Forward	TGCGCTCAGCAAACATTTATTG
	Reverse	CCAGTGCAGGGTCCGAGGTATT
U6	Forward	CTCGCTTCGGCAGCACATATACTA
	Reverse	ACGAATTTGCGTGTCATCCTTGCG

### Western Blotting

Total proteins were extracted with ice-cold RIPA lysis buffer (Solarbio, Beijing, China) plus PMSF (Solarbio) from ESCC cell lines. Then the protein concentrations were detected by a BCA assay kit (Solarbio). The separated proteins were isolated in 10% sodium dodecyl sulfate-polyacrylamide gel electrophoresis (SDS-PAGE), and transferred into PVDF membranes. After blocked by skim milk, these bands were incubated with primary antibodies against Bcl-2, Bax, cleaved caspase-3, cleaved caspase-9, MT1Mβ-actin (all from Abcam, Cambridge, United Kingdom) at 4°C overnights. Next, membranes were washed with PBS followed by incubation with the appropriate secondary antibody for 1 h at 22–23°C. Finally, the enhanced chemiluminescence (ECL, Pierce, Rockford, IL) visualized these membranes. Image J software was used to analyze membranes.

### CCK-8 Assay

The proliferation of ESCC cells was detected by CCK-8 assay. In brief, ESCC cells were seeded in the 96-well plate. Next, CCK-8 reagent was added to each well. The optical density (OD) value at 450 nm was measured by an enzyme plate analyzer (Shimadzu, Kyoto, Japan).

### Clone Formation Assay

Logarithmic growth cells (1 × 10^2^ cells/well) were inoculated into a 6-well plate and incubated for 7 days at 37°C in a 5% CO_2_ incubator. Then, 4% paraformaldehyde was fixed at room temperature for 30 s, and 0.1% crystal violet solution was dyed for 15 min. Finally, the colony formation number and relative colony formation were calculated by randomly selecting 10 fields under the microscope (Optical-SH, Shanghai, China).

### Flow Cytometry

The apoptosis of ESCC cells was assessed by flow cytometry assay. In brief, ESCC cells were collected and suspended in 100 μL of buffer solution. The cells were stained with FITC Annexin V and Propidium Iodide (PI) in dark for 10 min. Afterward, 400 μL of binding buffer was added to each tube, and the apoptosis was assessed by flow cytometry (BD Bioscience, CA, United States).

### Bioinformatics Analysis

The online prediction tool StarBase^1^ was used for the targeted prediction between LINC00261 and miR-545-3p, whereas Targetscan^2^ was used for the targeted prediction between miR-545-3p and MT1M.

### Luciferase Reporter Test

LINC00261-WT or LINC00261-Mut were co-transfected with miR-545-3p mimic or miR-NC into TE-1 and ECA109 cells using Lipofectamine 3000 (Thermo Fisher Scientific). MT1M-WT or MT1M-Mut were also co-transfected with miR-545-3p mimic or miR-NC into TE-1 and ECA109 cells using Lipofectamine 3000 (Thermo Fisher Scientific). After 48 h, the relative luciferase activities were detected by the dual Glo Luciferase Assay System (Promega, Shanghai, China) in accordance with the protocols. Renilla signals were used to normalize luciferase activity.

### Isolation of Nuclear and Cytoplasmic RNA

Nuclear and cytoplasmic RNA were separated using a Cytoplasmic and Nuclear RNA Purification Kit (Norgen Biotek, Canada) following the manuals of manufacturers. After purification and DNaseI treatment, the isolated nuclear or cytoplasmic RNA fractions were reverse transcribed and used for PCR as described above.

### Statistical Analysis

All data were expressed as mean ± SD. The Student’s *t*-test was used to determine the statistical differences between the two groups and ANOVA was used to analyze the statistical differences among the multiple groups. *P* < *0.05* was considered as statistically significant. All experiments were conducted in triplicates.

## Results

### LINC00261 Expression Is Decreased in ESCC Tissues and ESCC Cells

**FIGURE 1:**
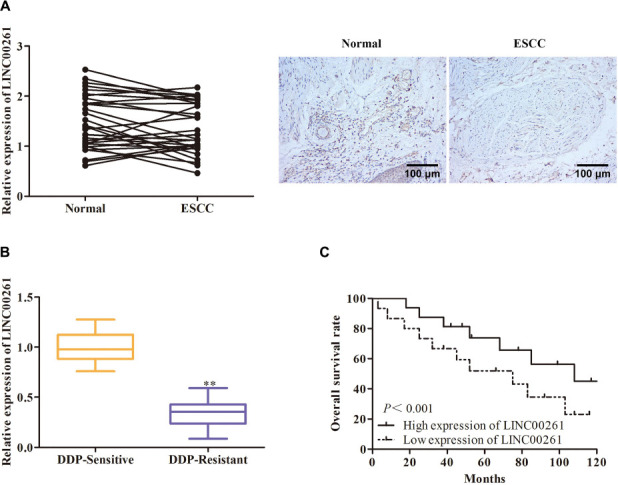
LINC00261 expression is decreased in ESCC tissues and ESCC cells. **(A)** The expression of LINC00261 in ESCC tissues and adjacent tissues. **(B)** The expression of LINC00261 in DDP-sensitive and DDP-resistant ESCC patients. **(C)** Survival analysis based on ESCC patients with high-level or low-level LINC00261. **P < 0.01 compared with the corresponding control group.

To determine whether LINC00261 affect ESCC, the expression of LINC00261 in ESCC tissues and adjacent tissue was first detected. The results showed that LINC00261 expression was significantly lower in ESCC tissues than in the normal adjacent tissues (Figure 1A). Interestingly, we found that the expression of LINC00261 was downregulated in DDP-resistant ESCC patients compared with DDP-sensitive ESCC patients (Figure 1B). Moreover, Kaplan-Meier survival analysis revealed that the high expression of LINC00261 was related to a high overall survival rate in ESCC patients (Figure 1C).

### Overexpression of LINC00261 Inhibits the Proliferation and Promotes Apoptosis of ESCC Cells

**FIGURE 2:**
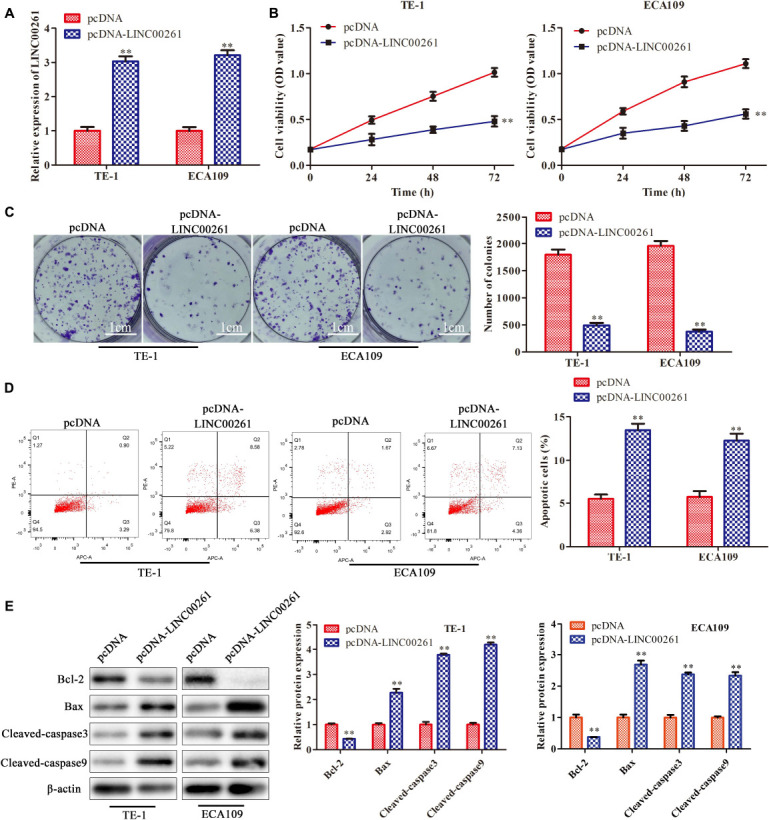
Overexpression of LINC00261 inhibits the proliferation and promotes apoptosis of ESCC cells. TE-1 and ECA109 cells were transfected with pcDNA-LINC00261 and pcDNA. **(A)** The expression of LINC00261 detected by RT-qPCR. **(B)** The proliferation assessed by CCK-8 assay. **(C)** The proliferation measured by clone formation assay. **(D)** The apoptotic cell detected by flow cytometry assay. **(E)** The apoptosis-relate protein detected by western blot in TE-1 and ECA109 cells. **P < 0.01 compared with the corresponding control group.

To explore the specific functions of LINC00261 in ESCC progression, pcDNA-LINC00261 and its corresponding control were transfected ESCC cells (TE-1 and ECA109). First, the transfection efficiency was detected by RT-qPCR and the results showed that LINC00261 expression was increased in the pcDNA-LINC00261 group compared to the control group (Figure 2A). CCK-8 and clone formation assay showed that overexpression of LINC00261 inhibited cell viability and reduced the number of colonies in TE-1 and ECA109 cells (Figures 2B,C), indicating that LINC00261 had a negative effect on ESCC cells proliferation. Besides, the apoptotic cells were remarkedly increased in the pcDNA-LINC00261 group compared with the control group (Figure 2D). Besides, western blot got similar results, which overexpression of LINC00261 induced Bax, cleaved-caspase 3, cleaved-caspase 9 expression and inhibited Bcl-2 expression in TE-1 and ECA109 cells (Figure 2E).

### Overexpression of LINC00261 Suppresses DDP Resistance in ESCC Cells

**FIGURE 3:**
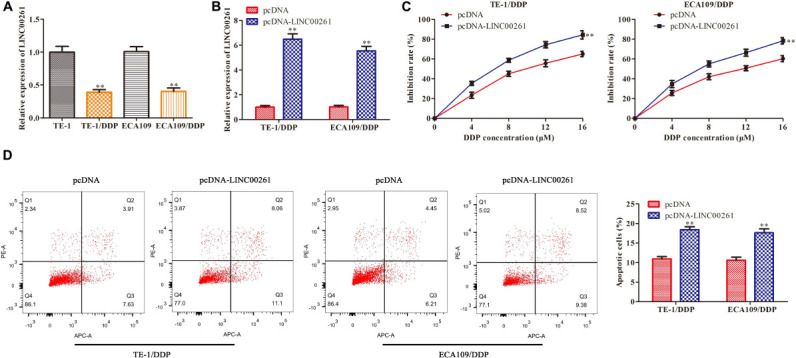
Overexpression of LINC00261 suppresses DDP resistance in ESCC cells. We constructed the DDP-resistant cells line (TE-1/DPP and ECA109/DDP). TE-1/DPP and ECA109/DDP were transfected with pcDNA-LINC00261 and pcDNA. **(A)** The expression of LINC00261 in TE-1, TE-1/DPP, ECA109, and ECA109/DDP cells. **(B)** The expression of LINC00261. **(C)** The proliferation inhibition rate assessed by CCK-8 assay. **(D)** The apoptotic cell detected by flow cytometry assay. **P < 0.01 compared with the corresponding control group.

To gain whether LINC00261 is involved in DDP resistance, we tested LINC00261 expression in TE-1, ECA109 and their corresponding DDP resistant (TE-1/DDP and ECA109/DDP) cell. As shown in Figure 3A, the expression of LINC00261 in TE-1/DDP (ECA109/DDP) cells was dramatically lower than that in TE-1 (ECA109) cells. To assess the functional role of LINC00261 in DDP resistance in ESCC cells, we overexpressed LINC00261 and evaluated its effect on cell proliferation and apoptosis in TE-1/DDP and ECA109/DDP cells. Overexpression of LINC00261 significantly promoted LINC00261 expression in TE-1/DDP and ECA109/DDP cells (Figure 3B). CCK-8 assay showed that cell proliferation was dose-dependently inhibited by DDP, overexpression of LINC00261 significantly inhibited the growth of DDP-supplemented cells compared with the control group (Figure 3C). Furthermore, flow cytometry assay revealed that overexpression of LINC00261 increased the apoptosis of TE-1/DDP and ECA109/DDP cells as compared with the pcDNA (Figure 3D).

### LINC00261 Functions as a Molecular Sponge for MiR-545-3p

**FIGURE 4:**
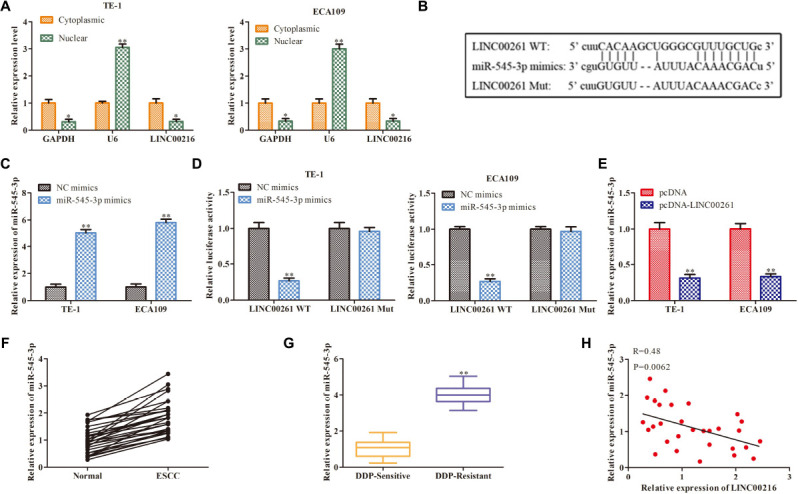
LINC00261 functions as a molecular sponge for miR-545-3p. **(A)** The location of NEAT1. **(B)** The binding sits between LINC00261 and miR-545-3p predicted by Bioinformatics websites. **(C)** The mRNA level of miR-545-3p in NC-mimics group and miR-545-3p mimics group. **(D)** The relationship between LINC00261 and miR-545-3p verified by luciferase reporter assay. **(D)** The mRNA level of miR-766-5p in PCa tissues. **(E)** The mRNA level of miR-545-3p in pcDNA group and pcDNA-LINC00261 group. **(F)** The expression of miR-545-3p in ESCC tissues and adjacent tissues. **(G)** The expression of miR-545-3p in DDP-sensitive and DDP-resistant ESCC patients. **(H)** Pearson’s correlation analysis demonstrated the correlation of LINC00261expression with miR-545-3p. *P < 0.05, **P < 0.01 compared with the corresponding control group.

Then we studied the specific mechanism of LINC00261 in ESCC. Numerous studies have shown that lncRNAs function as a competing endogenous RNA (ceRNA) binding to miRNA, then promote transcription (28281528). We firstly detected the cellular position of LINC00261 and found that LINC00261 was mainly located in the cytoplasm (Figure 4A). Bioinformatics prediction indicated that there were specific binding sites between the LINC00261 sequence and the miR−545-3p sequence, hinted that LINC00261 might be a target gene of miR−545-3p (Figure 4B). To confirm the relationship between LINC00261 and miR-545-3p, the luciferase reporter vectors LINC00261-WT or LINC00261-MUT, miR-545-3p mimics or NC mimics were constructed to transfect into TE-1 and ECA109 cells. MiR-545-3p mimic significantly prompted the expression of miR-545-3p (Figure 4C), and greatly reduced the luciferase activities of LINC00261-WT but had no effect on LINC00261-MUT (Figure 4D). In addition, RT-qPCR showed that overexpression of LINC00261 remarkably downregulated miR-545-3p (Figure 4E). Subsequently, we discovered that miR-545-3p expression was notably upregulated in ESCC tissues compared with adjacent tissues (Figure 4F), and the expression of miR-545-3p was increased in DDP-resistant ESCC patients compared with DDP-sensitive ESCC patients (Figure 4G). Besides, correlational analyses showed that there was a negative relationship between LINC00261 and miR-545-3p (Figure 4H). The above data demonstrated that LINC00261 functions as a sponger for the miR-5545-3p in ESCC.

### MiR-545-3p Promotes the Proliferation and Enhances DDP Resistance in ESCC Cells

**FIGURE 5:**
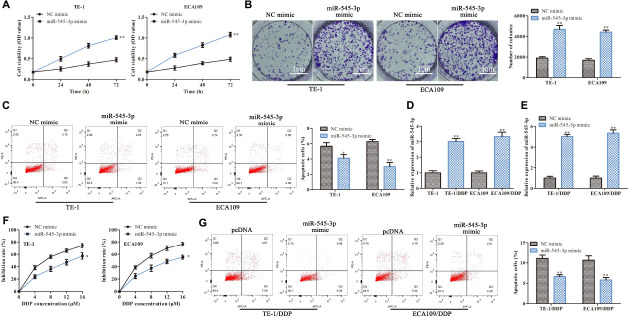
MiR-545-3p promotes the proliferation and enhances DDP resistance in ESCC cells. TE-1, ECA109, TE-1/DDP, and ECA109/DDP were transfected with miR-545-3p mimics and NC mimics. **(A)** The proliferation assessed by CCK-8 assay. **(B)** The proliferation measured by clone formation assay. **(C)** The apoptotic cell detected by flow cytometry assay. **(D)** The expression of miR-545-3p in TE-1, TE-1/DPP, ECA109, and ECA109/DDP cells. **(E)** The expression of miR-545-3p in TE-1/DPP and ECA109/DDP cells. **(F)** The proliferation inhibition rate assessed by CCK-8 assay. **(G)** The apoptotic cell detected by flow cytometry assay. *P < 0.05, **P < 0.01 compared with the corresponding control group.

Next, we further study the functions of miR-545-3p in the progression of ESCC. As shown in Figures 5A,B, CCK-8 and clone formation assay demonstrated that overexpression of miR-545-3p promoted the proliferation in TE-1 and ECA109 cells. Flow cytometry assay showed that the apoptotic rate was significantly reduced in the miR-454-3p mimics group compared with the NC mimics group (Figure 5C). On the other hand, we assessed the functional role of miR-545-3p in the DDP resistance of ESCC cells. RT-qPCR results showed that miR-545-3p expression in TE-1/DDP (ECA109/DDP) cells were vastly higher than that in TE-1 (ECA109) cells (Figure 5D). And miR-545-3p mimic promoted the expression of miR-545-3p whether in TE-1/DDP and ECA109/DDP cells (Figure 5E). Moreover, the CCK-8 assay showed that DDP inhibited cell proliferation in TE-1/DDP and ECA109/DDP cells dose-relatedly, overexpression of miR-545-3p significantly promoted the growth of DDP-supplemented cells compared with the control group (Figure 5F). Furthermore, flow cytometry assay revealed that overexpression of miR-545-3p decreased the apoptosis of TE-1/DDP and ECA109/DDP cells as compared with the pcDNA (Figure 5G).

### MT1M Is a Target Gene of MiR-545-3p

**FIGURE 6:**
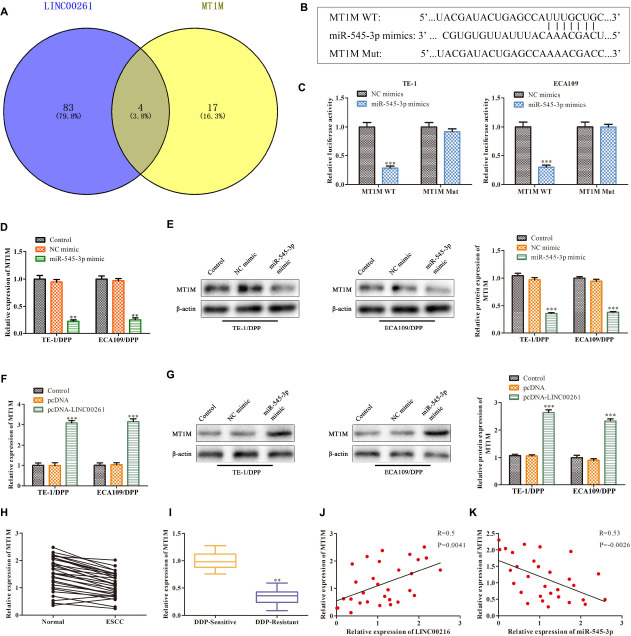
MT1M is a target gene of miR-545-3p. **(A)** The target gene of miR-545-3p predicted by Bioinformatics websites. **(B)** The binding sites between miR-545-3p and MT1M. **(C)** The interaction between miR-545-3p and MT1M testified by luciferase reporter assay. **(D)** The mRNA level of MT1M detected by RT-qPCR in miR-545-3p mimics treated cells. **(E)** The protein level of MT1M measured by western blot in miR-545-3p mimics treated cells. **(F)** The mRNA level of MT1M detected by RT-qPCR in pcDNA-LINC00261 treated cells. **(G)** The protein level of MT1M measured by western blot in pcDNA-LINC00261 treated cells. **(H)** The expression of MT1M in ESCC tissues and adjacent tissues. **(I)** The expression of MT1M in DDP-sensitive and DDP-resistant ESCC patients. **(J)** Pearson’s correlation analysis demonstrated the correlation of LINC00261expression with MT1M. **(K)** Pearson’s correlation analysis demonstrated the correlation of miR-545-3p expression with MT1M. **P < 0.01, ***P < 0.001 compared with the corresponding control group.

To study the downstream functional genes of miR-545-3p, the Bioinformatics website was used to predict that MT1M might be a target gene of miR-545-3p (Figure 6A), and there was a binding site between the MT1M sequence and miR-545-3p sequence (Figure 6B). Luciferase reporter analysis showed a significant reduction in the luciferase activity of the 3′UTR region of MT1M when the cells were co-transfected with MT1M WT and miR-545-3p mimics. However, there was no notable difference in luciferase activity of MT1M Mut between the miR-545-3p mimics group and the control group (Figure 6C). In addition, we found that the protein and mRNA levels of MT1M were significantly downregulated in the miR-545-3p mimic group compared with the NC mimics group (Figures 6D,E). Thereafter, we detect the relationship between LINC00261 and MT1M. RT-qPCR and western blot analysis showed that overexpression of LINC00261 upregulated MT1M expression (Figures 6F,G). And MT1M expression was downregulated in ESCC tissues compared to adjacent tissues (Figure 6H). Interestingly, the expression of MT1M was notably downregulated in DDP-resistant ESCC patients compared with DDP-sensitive ESCC patients (Figure 6I). Furthermore, correlation analysis confirmed a positive correlation between LINC00261 and MT1M (Figure 6J), while a negative correlation between miR-545-3p and MT1M (Figure 6K). These results demonstrated that MT1M is a downstream target gene of miR-545-3p.

### LINC00261 Inhibits ESCC Progression and DDP Resistance by MiR-545-3p/MT1M Axis

**FIGURE 7:**
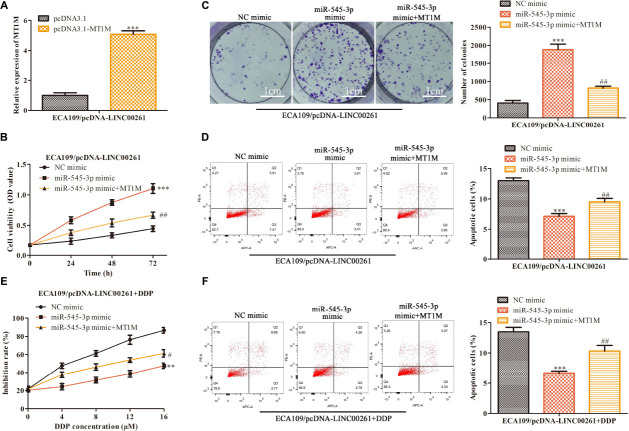
LINC00261 inhibits ESCC progression and DDP resistance by miR-545-3p/MT1M axis. MT1M, miR-545-3p and their corresponding control were used to transfect the stable overexpression LINC00261 cell line (ECA109/pcDNA-LINC00261). **(A)** The expression of MT1M detected by RT-qPCR. **P < 0.01, ***P < 0.001 compared with the corresponding control group. **(B)** The proliferation assessed by CCK-8 assay. **(C)** The proliferation measured by clone formation assay. **(D)** The apoptotic cells detected by flow cytometry assay. **(E)** The proliferation inhibition rate assessed by CCK-8 assay. **(F)** The apoptotic cell detected by flow cytometry assay. **P < 0.01, ***P < 0.001 compared with the NC-mimic group, ^#^*P* < 0.05, ^##^*P* < 0.01 compared with the miR-535-3p-mimic group.

Finally, attempts were made to determine whether LINC00261−mediated effects were indeed through miR-545-3p/MT1M axis, MT1M, miR-545-3p, and their corresponding control were used to transfect the stable overexpression LINC00261 cell line (ECA109/pcDNA-LINC00261). The transfection efficiency was measured by RT-qPCR and the result was showed in Figure 7A. CCK-8 and clone formation assay suggested that overexpression of MT1M partly reversed the positive effects of miR-545-3p mimics on the proliferation in ECA109/pcDNA-LINC00261 cells (Figures 7B,C). Compared with the miR-545-3p mimics group, increased apoptotic cells were detected in the miR−545-3p mimics + MT1M group (Figure 7D). Additionally, overexpression of MT1M reversed the effects of miR-545-3p mimics on the growth and the apoptosis in DDP-supplemented ECA109/pcDNA-LINC00261 cells (Figures 7E,F).

## Discussion

In the current study, LINC00261 expression decreased in ESCC tissues and ESCC cells. It was also found that overexpression of LINC00261 inhibited cell proliferation and promoted cancer cell apoptosis. Further, LINC00261 could suppressed the DDP resistance of ESCC and it participate in regulation through miR-545-3p/MT1M axis. DDP is a common clinical chemotherapy drug, which induces apoptosis of G2 phase cells to achieve the killing effect on malignant tumor cells (Ferreira et al., 2016). It is widely used in the treatment of a variety of tumors including ESCC. However, low-concentration DDP is very easy to cause drug resistance, which limits its wide clinical application. At the same time, DDP-resistant also severely affects the actual curative effect of DDP, and eventually causes recurrence of ESCC patients after postoperative chemotherapy, leading to chemotherapy failure (Sharma et al., 2012). Therefore, DDP resistance has become an important factor that seriously affects the prognosis of patients with ESCC. At present, the mechanism of DDP-resistance in ESCC has not been fully explored. Hence, a better understanding of the molecular mechanism of cisplatin resistance in ESCC is needed to improve the prognosis.

Recent studies have revealed that certain lncRNAs are involved in drug resistance (Song et al., 2019; Tian et al., 2019; Wu et al., 2019). Furthermore, the gene LINC00261 was determined by comparing the expression of lncRNAs in ESCC and adjacent tissues. Interestingly, lower levels of LINC00261 were also observed in DDP-resistant ESCC patients compared with DDP-sensitive ESCC patients. In view of these findings, LINC00261was first confirmed to exert a major role in the formation of cisplatin resistance.

The previous study has revealed that lncRNAs contribute to transcriptional and posttranscriptional regulation in ESCC. For instance, Yao et al. (2019) found that lncRNA MALAT1 was significantly upregulated in the ESCC cells, and MALAT1 increased the stemness of ESCC by enhancing YAP transcriptional activity. The progression of DDP-resistant in tumors was related to abnormal expression of lncRNA. For example, overexpression of FOXD2-AS1 enhanced DDP-resistance in ESCC tissues and cells, and silencing FOXD2-AS1 improved DDP-resistance in ESCC (Liu H. et al., 2020). LINC00261 acts as a tumor suppressor gene in many cancers, LINC00261 suppresses the growth and metastasis of liver cancer, prostate cancer, and pancreatic cancer (Guo et al., 2020; Li Y. et al., 2020; Liu et al., 2020). It has been stated in the results that LINC00261 is the candidate gene screened in our previous experiments. Furthermore, overexpression of LINC00261 relieves DDP-resistance of colon cancer cells by increasing cell apoptosis and inhibiting proliferation (Wang et al., 2017). All these reports demonstrated that LINC00261 could be a potential target for the treatment of cancer. Our study showed that LINC00261 was down-regulated in the ESCC tissues and cells, as well as in patients with ESCC that are resistant to DDP. Additionally, patients with higher expression of LINC00261 had a longer overall survival time. Further experiments revealed that overexpression of LINC00261 remarkedly inhibited the proliferation, DDP-resistant, and promoted apoptosis of ESCC cells. These results further support the idea of that LINC00261 may be a key therapeutic target for ESCC.

Increasing reports demonstrated that lncRNAs took effects on tumorigenesis by bonding with specific miRNA. In order to dig further and find the combined miRNA, a series of follow-up studies will be carried out. In this study, LINC00261 was mainly located in the cytoplasm of ESCC cells by subcellular separation assay, illustrating that LINC00261 might modulate its downstream gene expression after transcription. Bioinformatics websites predicted the target gene of LINC00261 and identified miR-545-3p which has been reported to exert major roles in multiples cancers, such as non-small-cell lung cancer cells (Li H. et al., 2020), hepatocellular carcinoma (Changjun et al., 2018), and laryngeal cancer (Cui et al., 2019). Our present study verified that LINC00261 served as a ceRNA for miR-545-3p and negatively regulated miR-545-3p expression. In addition, miR-545-3p expression was greatly increased in ESCC tissues and cells, as well as in patients with ESCC that are resistant to DDP. And miR-545-3p promoted the proliferation and enhanced DDP resistance in ESCC cells. These results are in line with those of previous studies.

Thereafter, we further explored the mechanisms of miR-545-3p on ESCC progression. Bioinformatics websites predicted the target gene of miR-545-3p, identified MT1M. MT1M is a cysteine-enriched small molecule protein, belonging to the metallothionein (MT-1) family. MTs are involved in the regulation of tumor cell growth, angiogenesis, metastasis, and drug resistance, and MTs family members have different anti-cancer or anti-cancer effects in different types of tumors (Si and Lang, 2018). In hepatocellular carcinoma, the low expression of MT1M is positively correlated with poor prognosis, suggesting that MT1M plays a tumor-suppressive role in hepatocellular carcinoma. However, there are few reports on the function of this family of genes in ESCC. In this study, MT1M was downregulated in ESCC tissues and cells, as well as in patients with ESCC that are resistant to DDP, and was positively correlated with LINC00261 whereas was negatively correlated with miR-545-3p. And rescue experiments demonstrated that MT1M overexpression reversed that miR-545-3p mimic-induced positive function on the proliferation and DDP-resistant in ESCC cells. This finding fills the gap in the molecular regulation mechanism of MT1M in ESCC and is helpful to further improve the prognostic treatment of ESCC.

In summary, the findings demonstrated a potential therapeutic target for ESCC and found the molecular mechanism of action. LINC00261 regulated the proliferation and apoptosis of ESCC, further suppressed the DDP resistance of ESCC by miR-545-3p/MT1M axis.

## Data Availability Statement

The raw data supporting the conclusions of this article will be made available by the authors, without undue reservation.

## Ethics Statement

The studies involving human participants were reviewed and approved by the Ethics Committee of Jiangsu Cancer Hospital. The patients/participants provided their written informed consent to participate in this study.

## Author Contributions

LW, XJ, and YL drafted the manuscript, conceived and designed the study, and accomplished the revision of manuscript for important intellectual content. XW performed the acquisition of data. PY performed the analysis and interpretation of data. LW and YL obtained funding. All authors read and approved the final manuscript.

## Conflict of Interest

The authors declare that the research was conducted in the absence of any commercial or financial relationships that could be construed as a potential conflict of interest.

## Footnotes

1 http://starbase.sysu.edu.cn/

2 http://www.targetscan.org/vert_72/
